# Region- and layer-specific investigations of the human menisci using SHG imaging and biaxial testing

**DOI:** 10.3389/fbioe.2023.1167427

**Published:** 2023-04-18

**Authors:** Bismi Rasheed, Venkat Ayyalasomayajula, Ute Schaarschmidt, Terje Vagstad, Hans Georg Schaathun

**Affiliations:** ^1^ Cyber-Physical Systems Laboratory, Department of ICT and Natural Sciences, Norwegian University of Science and Technology (NTNU), Ålesund, Norway; ^2^ Ålesund Biomechanics Lab, Ålesund General Hospital, Møre and Romsdal Hospital Trust, Ålesund, Norway; ^3^ Division of Biomechanics, Department of Structural Engineering, Norwegian University of Science and Technology (NTNU), Trondheim, Norway; ^4^ Department of Orthopaedic Surgery, Medi3, Ålesund, Norway

**Keywords:** human menisci, constitutive modeling, biaxial testing, second harmonic imaging, multi-photon microscopy, collagen fiber, fiber distribution, surgery simulation

## Abstract

In this paper, we examine the region- and layer-specific collagen fiber morphology via second harmonic generation (SHG) in combination with planar biaxial tension testing to suggest a structure-based constitutive model for the human meniscal tissue. Five lateral and four medial menisci were utilized, with samples excised across the thickness from the anterior, mid-body, and posterior regions of each meniscus. An optical clearing protocol enhanced the scan depth. SHG imaging revealed that the top samples consisted of randomly oriented fibers with a mean fiber orientation of 43.3^*o*^. The bottom samples were dominated by circumferentially organized fibers, with a mean orientation of 9.5^*o*^. Biaxial testing revealed a clear anisotropic response, with the circumferential direction being stiffer than the radial direction. The bottom samples from the anterior region of the medial menisci exhibited higher circumferential elastic modulus with a mean value of 21 MPa. The data from the two testing protocols were combined to characterize the tissue with an anisotropic hyperelastic material model based on the generalized structure tensor approach. The model showed good agreement in representing the material anisotropy with a mean *r* ^2^ = 0.92.

## 1 Introduction

The meniscus is a crescent-shaped tissue present between the femoral condyle and the tibial plateau of the human knee joint. This fibrocartilaginous soft tissue plays a crucial role in load distribution and structural stability. It also acts as a shock absorber and provides lubrication and nutrition to the knee joint (Makris et al., 2011; Fox et al., 2012). The human knee joint has two menisci; the C-shaped medial meniscus is on the inner side of the knee joint, and the O-shaped lateral meniscus is on the outer side of the knee. The medial meniscus is restricted in its range of motion and more susceptible to injury than the lateral meniscus, as it is tightly attached to the knee joint capsule (Lento and Akuthota, 2000). This difference, along with the shape of the menisci, and variation in applied loading, contributes to the *anatomical location specificity* in material properties. When viewed from above, three distinct regions can be identified in both menisci. The dissimilarity of width in these anterior, mid-body, and posterior regions together with differing load distributions, produces various stress concentrations, which result in the *region-specific* material properties (Danso et al., 2017). In the cross-sectional view, the topmost layer, so-called the superficial layer, is characterized by randomly arranged fibers. Lamellar collagen fibrils make up the second layer, referred to as the lamellar layer. The central main layer, underneath the lamellar layer, has predominant bundles of circumferentially arranged fibers (Fithian et al., 1990; Petersen and Tillmann, 1998; Fox et al., 2012). This depth-wise heterogeneity in fiber quantity and fiber orientation controls the *layer-specific* material properties. The anisotropic tissue behavior resulting from this region- and layer-specificity must be incorporated into material models for accurate biomechanical characterization.

The formulation of tissue mechanical behavior led to the development of constitutive models, which are mathematical frameworks based on fundamental physical principles. These models aid in understanding structure-function relationships and, as a result, help predict the mechanical response of the tissue to different loading (Imeni et al., 2020). These are valuable in medical applications such as tissue engineering and the design of synthetic replacement alternatives (Kahlon et al., 2015). The awareness of biomechanical function also helps in studying the meniscus injury mechanism, which leads to the selection of suitable repair strategies (Lento and Akuthota, 2000). Additionally, the computational models of the knee joint, including the constitutive framework of the menisci, assist in assessing the impact of meniscus geometry (Luczkiewicz et al., 2018), meniscal tears, meniscectomy, and osteoarthritis (Li et al., 2020) on the biomechanics of the knee joint. The fidelity of these knee joint models is tied directly to the constitutive model parameters, which must be well characterized by the mechanical response of the material (Morrow et al., 2010). For this, mechanical testing has commonly been the gold standard.

Numerous studies have reported region-wise differences in the material properties of the meniscus, but only a few have focused on layer-wise mechanical characterization. The indentation test exhibited higher elastic moduli (Danso et al., 2015; Gaugler et al., 2015; Danso et al., 2017), higher strain-dependent fibril network modulus (Danso et al., 2017), and higher permeability (Danso et al., 2017) in the anterior horn of the medial menisci. However, estimating anisotropic behavior and developing a mathematical model from indentation test data is extremely challenging (Navindaran et al., 2023). Unconfined compression testing on human menisci showed a significantly lower compressive modulus than the tensile modulus (Chia and Hull, 2008). In contrast to the direct correlation of the tensile characteristics with the collagen orientation (Bursac et al., 2006), it has little effect on the compressive moduli (Leslie et al., 2000; Chia and Hull, 2008). Uniaxial tensile testing on human menisci revealed the greater circumferential tensile modulus (Lechner et al., 2000) as well as higher elastic and viscous dynamic moduli (Bursac et al., 2006) of the anterior region of the medial menisci. In addition to the region-specificity implied in these studies, the variation of elastic and viscous properties across the meniscus (which indicates layer-specificity) was demonstrated using uniaxial testing on porcine menisci (Maritz et al., 2021). However, stretching the tissue fibers in the uniaxial direction causes compression of the fibers in the lateral directions, which is undesirable. This can be overcome by biaxial testing, which can better replicate the complex *in vivo* loading conditions (Jacobs et al., 2013). Kahlon et al. (2015) studied regional and depth variability of porcine meniscus properties through biaxial testing, and noticed higher Young’s moduli in the layers near to femur and tibia. Understanding this location-specific material behavior is crucial for comprehending the macroscopic behavior of the meniscus and developing appropriate constitutive models (Barrera et al., 2018).

The constitutive models that do not incorporate information from fiber families are not able to capture the stress-strain response of different layers accurately (Gasser et al., 2005). Many imaging modalities have been used to quantitatively describe the three-dimensional structural morphology of collagen fibers at the micro-nano scale. Light microscopy (Andrews et al., 2014; Bonomo et al., 2020), electron microscopy (Fithian et al., 1990), scanning electron microscopy (SEM) (Vetri et al., 2019), micro-computed tomography (*μ*CT) (Agustoni et al., 2021), and multiphoton microscopy (MPM) (Vetri et al., 2019; Sancataldo et al., 2022) studies elucidated the complex inhomogeneous architecture of the human meniscal tissue. According to a prior spectroscopic investigation (Danso et al., 2017), the anterior region of the medial menisci has a larger collagen content, which explains the higher elastic moduli reported in many studies. In addition to this, they observed a lower collagen orientation angle at the top layer of the menisci. Collagen fibers in the layers adjacent to the femoral condyle and tibial plateau were randomly aligned in *μ*CT investigations of the mid-body of the lateral meniscus, whereas collagen fibers with a 30° alignment were observed in the deep interior layer (Agustoni et al., 2021). Maritz et al. (2021) demonstrated that this random alignment of the collagen fibers in the superficial layers results in stiffer mechanical responses compared to internal regions. These investigations are all consistent in showing the heterogeneity of the meniscus in a width-wise (region-specificity) and depth-wise (layer-specificity) manner. However, deep imaging in complex environments is an important challenge in these imaging modalities. The imaging modality used in identifying region-and layer-specific collagen details should be capable of producing ultra-high resolution images deep within optically thick samples. Multiphoton microscopy (Williams et al., 2001) is well-suited for this purpose. In addition, second harmonic generation (SHG) (Freund and Deutsch, 1986) enables direct imaging of collagen fibers. These imaging techniques can be employed simultaneously to image intrinsic tissue indicators like collagen, which have a high degree of hyperpolarizability (Zipfel et al., 2003). MPM and SHG studies on the human meniscus, for example, have shown that collagen bundles are stacked in compartments with a honeycomb-like pattern in the cross-section of the meniscal tissue (Vetri et al., 2019). Another intriguing observation achieved utilizing MPM on the bovine meniscus was the arborization of “tie-fibers” and its association with circumferential fibers (Andrews et al., 2014). SHG imaging is also an effective tool for diagnosing osteoarthritis, even in its early stages, by analyzing the thickness and organization of collagen fibers in different layers (Hui Mingalone et al., 2018). Therefore, in this paper, we employed an SHG via MPM-enabled imaging of collagen fiber morphology to obtain data for constitutive modeling.

A wide variety of numerical models of the meniscus tissue have been reported, ranging from simple isotropy to complex anisotropy consideration (Imeni et al., 2020; Elmukashfia et al., 2022). Previous mechanical studies suggested a plane of isotropy for the material properties (Chia and Hull, 2008), which led to models that took transverse isotropy into account (Spilker et al., 1992; LeRoux and Setton, 2002). Fibril-reinforced modeling has been included in a few studies (LeRoux and Setton, 2002; Abraham et al., 2011; Danso et al., 2015; Danso et al., 2017; Seyfi et al., 2018). Freutel et al. (2015) and Seyfi et al. (2018) presented a constitutive model that takes the local collagen network into account based on the strain energy density (SED) function proposed by Holzapfel et al. (2000). Both of these studies used the inverse finite element method to determine the regional variation in material properties. Despite having the advantage of allowing materials to be tested non-invasively while retaining *in vivo* conditions, this technique has the disadvantage of requiring a large number of simulations (Bäker and Shrot, 2013). Abraham et al. (2011) presented an anisotropic hyperelastic model based on circumferential and radial fibers in different regions, however, the samples were extracted only from the central main layer, indicating a lack of layer specificity. Moreover, all of the aforementioned constitutive models have been on animal menisci. For a detailed review of the available constitutive models applied to the meniscus tissue and their sensitivity analysis, the reader is redirected to Elmukashfia et al. (2022).

The observations from mechanical testing and histology examinations confirm variations in the mechanical behavior of the different layers within the regions of the menisci, suggesting the significance of defining distinct region- and layer specificity as well as taking direction dependence into account when modeling the meniscus (Seyfi et al., 2018). The constitutive model based on this data would be advantageous in total knee simulations, which require the capture of the highly complex material behavior of the meniscus with high accuracy. This study is a part of developing a digital twin for human knee joint for practicing arthroscopic surgery (Bjelland et al., 2022). Probing meniscus is a common task in meniscectomy to ascertain the tissue characteristic as being hard or soft (Hu and Desai, 2004), which usually serves as the initial guide for surgeons to diagnose degenerative meniscus (Anetzberger et al., 2014). Hence, the quasi-static deformation must be modeled accurately for providing accurate haptic feedback to the surgeon in surgical training and simulation (Tuijthof et al., 2011). The material model for the meniscus used in the digital twin must be realistic enough to replicate the differing deformation in each layer within the region during the *in-vivo* probe-meniscus interaction, as the success of pre-operative planning hinges on every aspect of the surgical simulation. In this regard, a constitutive framework for human menisci is presented here based on the strain energy density function proposed by Holzapfel et al. (2015). The mechanical characterization is done by *in situ* biaxial tensile testing in different layers of each region of the lateral and medial menisci. As our study focused on quasi-static behavior, special attention was given to minimizing the viscoelastic and poroelastic effects. The dispersed collagen orientation is characterized by multi-photon microscopy based on second harmonic imaging. Finally, the material data obtained from these two approaches were incorporated into the constitutive equation. The results help in identifying the anisotropy of different tissue layers in different regions in both menisci. This region- and layer-specific constitutive model aids surgeons in better decision-making and the design of more realistic meniscal prostheses. The paper is organized as follows: The methods section provides an overview of the experimental setup and post-processing of the obtained data. The results section demonstrates novel findings in terms of both collagen organization, regional non-linear anisotropic mechanical response, and structure-function relationships. The discussion section presents the main contributions, comparisons to existing literature, and limitations of the current study. Finally, the overall conclusions and future perspectives are presented.

## 2 Methods

### 2.1 Sample preparation and storage

**FIGURE 1:**
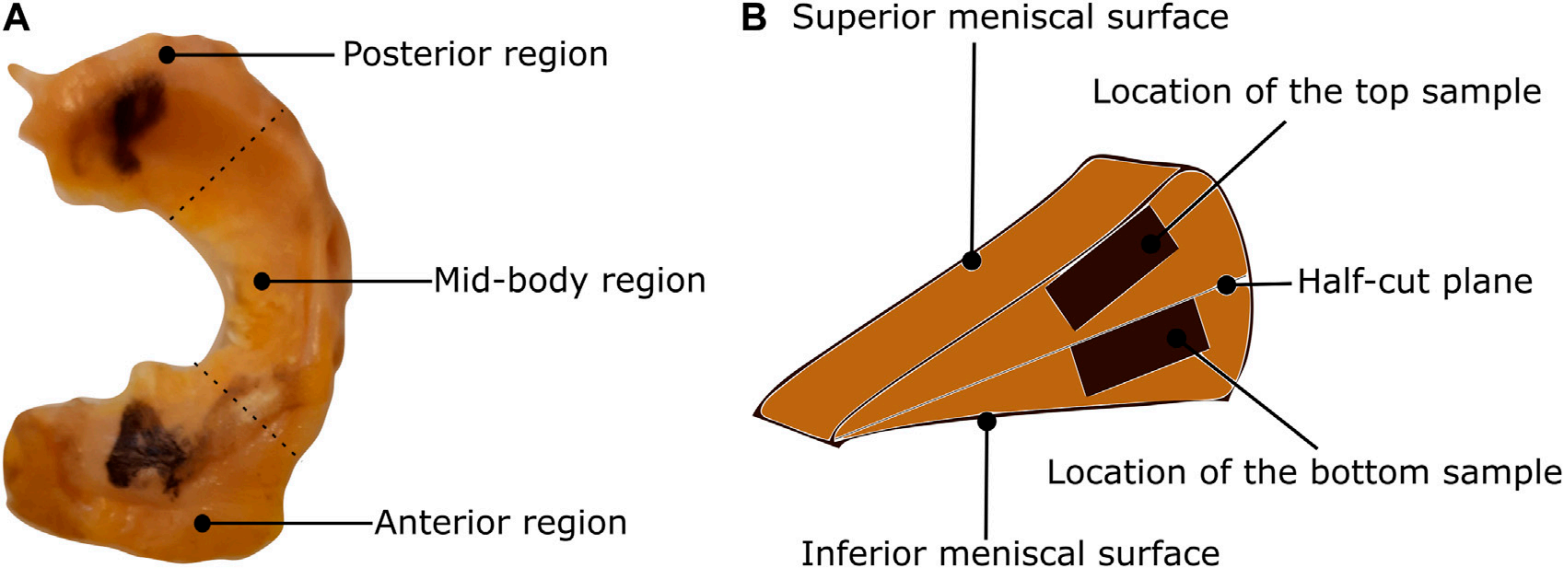
Classification of the meniscus tissue into three regions and two layers **(A)** The top view of the lateral meniscus of the left knee with identification of the posterior, mid-body, and anterior regions. **(B)** The location of the samples on the cross-sectional view.

In this study, a total of nine human menisci were examined including five lateral menisci and four medial menisci. The tissues were procured from human cadavers with an average age of 75 years. The research was approved by Regionale komiteer for medisinsk og helsefaglig forskningsetikk (REK midt), Trondheim, Norway dated 27.01.2021 with reference number 214114. This study has followed the ethical principles for medical research involving human subjects outlined in the World Medical Association’s Declaration of Helsinki. The tissues were transported in an ice chamber to minimize any degradation during transport. Once in the laboratory, they were stored in a freezer operating at −28*°*C. Brief storage of soft tissues in this freezing condition was shown to have minimal effects on the observed biaxial mechanics in previous studies (Duginski et al., 2020). On the day of mechanical testing, the tissues were thawed in phosphate-buffered saline (PBS) solution at room temperature (20*°*C). To prepare repeatable samples, the following slicing procedure is used: First, the circumferential length is measured using a thread from the tip of the anterior region to the end of the posterior region. Then, the meniscus is sliced into three regions based on one-third of the circumferential measurement (Figure 1A) using a surgical knife. Next, each region is cut into halves (the top half layer and the bottom half layer) using a half-cut plane, as shown in Figure 1B. Finally, the *top sample* of dimension 10 × 10 mm is extracted from the top half layer, starting from the superior meniscal surface (the surface near the femoral condyle) and extending to around 2 mm (2.1 ± 0.1 mm) normal to it. In the same way, the *bottom sample* is extracted from the bottom half layer but starting from the half-cut plane. This is to ensure the top sample is representative of a combined superficial and lamellar layer and the bottom sample is from the central-main layer. The sample thickness was ensured to be constant within the central region (2 mm), however, it was intricate to ensure uniform thickness for the whole sample using a surgical knife. A better method could be the protocol presented in Bonomo et al. (2020). In total, six samples were tested per meniscus to quantify their mechanical behavior.

### 2.2 Biaxial mechanical testing

**FIGURE 2:**
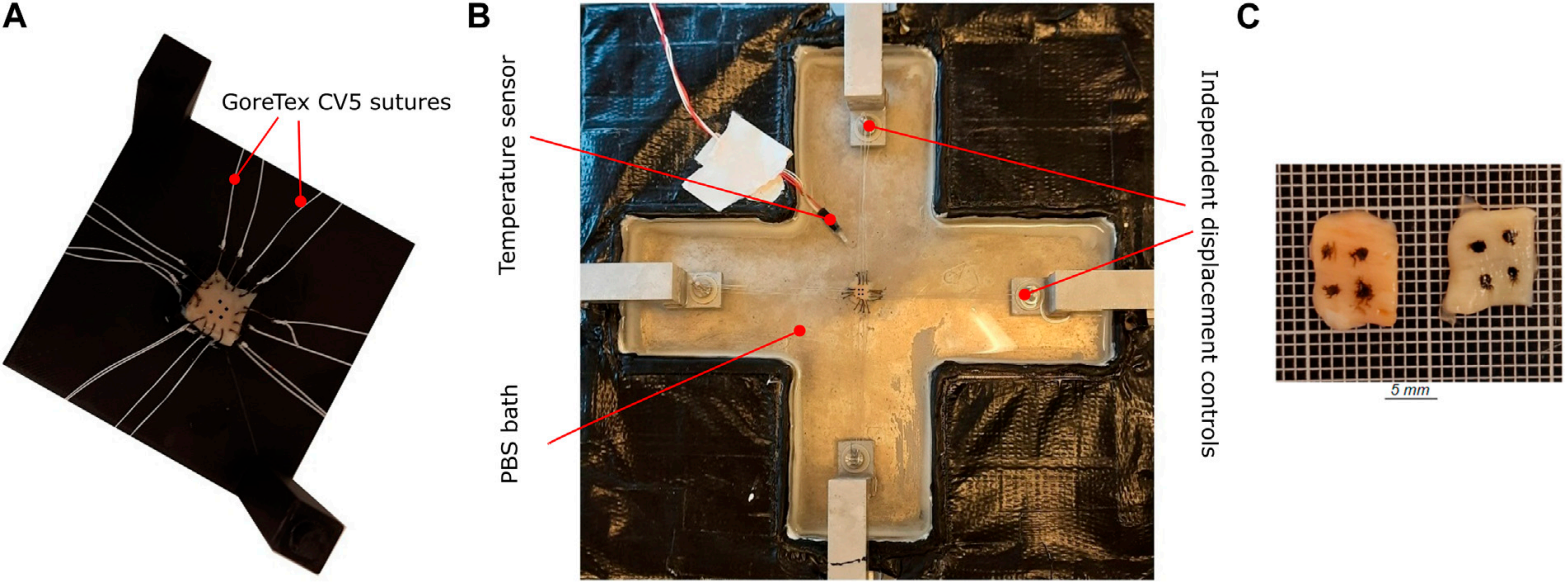
Sample preparation and mounting protocol for biaxial tension testing **(A)** The sample is placed on a 3D-printed template. The sample is attached with barbless hooks and sutures, marked using tissue marking dye. **(B)** Custom-built biaxial tensile test set-up. The sample is submerged in PBS. **(C)** The sample after optical tissue clearing.

Once the menisci samples were thawed, they were marked with indicative markers using a tissue marking dye (Leica Biosystems, Germany). This ensures a proper distinction between the circumferential and radial directions during testing and imaging. The sample thickness was then measured three times with a caliper (0.01 mm resolution), whereby the average value is reported. Finally, the sample was attached to four barbless hooks (Ahrex, Denmark) on each side, using a 3D-printed template for consistency. The 10 × 10 mm sample was additionally marked with four fiducial markers in the center, with an approximate area of 2 × 2 mm, as shown in Figure 2A. The samples were submerged in a PBS bath heated to 36*°*C for the entire duration of the biaxial mechanical test. Doing so ensures that the sample is hydrated in an isotonic solution that mimics the *in-vivo* conditions and minimizes fluid leakage/drying up of the tissue. The hooks are connected to the handle mechanism with surgical Gore-Tex CV5 sutures (Gore Medicals, United States). The custom-designed handle mechanism consists of a rotatable base with two 1 mm thick bars representing the sutures’ attachment sites. These bars can rotate freely around the base axis, which is equipped with an all-ceramic bearing (SMB Bearings, United Kingdom). This mechanism enables self-alignment when mounting the sample and minimizes the shear forces during the experiments (Sommer et al., 2015). The custom-built biaxial machine (see Figure 2B) is equipped with four step motors that are individually controlled via an inductive displacement sensor (BAW M18MG, Balluff, Germany). The loads are also measured with an axial load transducer (U9C/50 HBM, Germany). Using a custom software for digital image correlation (Fagerholt et al., 2013), the fiducial markers are tracked during the test and synchronized with the measured forces at a frequency of 2 *Hz*. Before starting the test, preconditioning of five loading-unloading cycles at a rate of 0.1 mm/s was applied up to a stretch of 1.30. Additionally, the sample was subjected to a preload of 0.01 N in both directions to remove any tissue slack. Three sets of displacement-controlled tests, each repeated three times, were carried out at a speed of 0.1 mm/s up to the maximum load 1 N. The total loading cycle varied from sample to sample with an average time of 40 s. The chosen quasi-static loading rate ensures that the measured mechanical response corresponds to the passive-elastic properties of the tissue, with minimal relaxation and creep effects (Wang et al., 2018). However, pausing the test momentarily for microstructure imaging for instance would affect the stress-stretch response as seen in Pukaluk et al. (2023). Sufficient care was taken in the design of the experiment to only measure the elastic response of the tissue and minimize any relaxation effects. Further, the damping effect has not been measured using cyclic testing. The displacement configurations used in the three test sets represent a different displacement ratio of circumferential to radial direction (1 : 1, 1 : 1.2, 1.2 : 1). The acquired force and deformation data are then used to compute the stress-stretch curves of the sample.

### 2.3 Optical tissue clearing

The same samples that were subjected to biaxial tension were chemically fixed prior to the microscopy investigations. Samples were chemically fixed in a 4% formaldehyde solution for a minimum of 36 h and also dehydrated based on graded absolute ethanol: 50%, twice 70%, twice 95%, and twice 100% with each step lasting 30 min (Schriefl et al., 2013). The fixation and dehydration were performed in an automated vacuum tissue processor (ASP 300, Leica Biosystems, Germany). Once the tissue sample was chemically fixated, it was then subjected to an optical clearing protocol called SeeDB (Ke et al., 2013). The tissue samples were treated in graded solutions of fructose: 4–8 in 20%, 4–8 in 40%, 4–8 in 60%, 12 in 80%, 12 in 100%, and 24 h in SeeDB solution. The solutions were maintained at room temperature while being mixed uniformly on a tube rotator for the entire duration of the clearing. A representative cleared sample is shown in Figure 2C.

### 2.4 Second harmonic generation imaging

SHG imaging of collagen fibers was performed using a Leica SP8 confocal microscope (Leica Microsystem, Germany). Before the SHG acquisition, the measurement of the entire thickness was verified with the bright field microscope in the Leica SP8 to ensure that the SHG scanning occurs through the entire thickness of the samples. To induce the second harmonic of collagen fiber, the laser excitation was set to 890 nm. The Leica HCX IRAPO 25×, NA 0.95 water objective with a working distance of 2.4 mm was used for image acquisition. To compensate for scattering at greater depth, the laser power was increased linearly (z compensation). The scanning took place at the center of the sample with an area of 465 × 465 *μ*m and a resolution of 0.45 *μ*m/pixel from the top side (superior). Imaging was performed at 2 *μ*m intervals across the entire thickness of the sample. The optically cleared samples were imaged up to a depth of 500 *μ*m with an excellent resolution of the collagen fiber network, compared to around 150 *μ*m for non-cleared tissue samples.

### 2.5 Collagen fiber morphology quantification

A user-defined Matlab script based on the method documented in Polzer et al. (2013) is used to quantify the collagen fiber dispersion in each layer. Prior to the analysis, a 2D Tukey (tapered cosine) window was applied to the image. Each image was then Fourier transformed and multiplied by its complex conjugate to form the power spectral density, which distinguishes the fiber direction by frequency and orientation. A wedge-shaped filter was used to extract the fiber orientations at a certain angle *θ*. The wedge-shaped filter ranges from −89.5° to 89.5° with an increment of 1° to determine the relative amplitude of the fiber distribution. A moving average filter with a range of 5° was applied to the angle *θ* to smooth the data. For a certain anisotropic fiber morphology, the fiber distribution is then fitted by a von Mises probability distribution as defined in Eq. 1 and Eq. 2.

$$f\left(\theta |\mu ,a\right)=\frac{e^{a\;cos2\left(\theta -\mu \right)}}{\pi I_{0}\left(a\right)}$$ (1)

$$I_{0}\left(a\right)=\frac{1}{\pi }\int_{0}^{\pi }e^{a\;\cos\theta }d\theta$$ (2)

**FIGURE 3:**
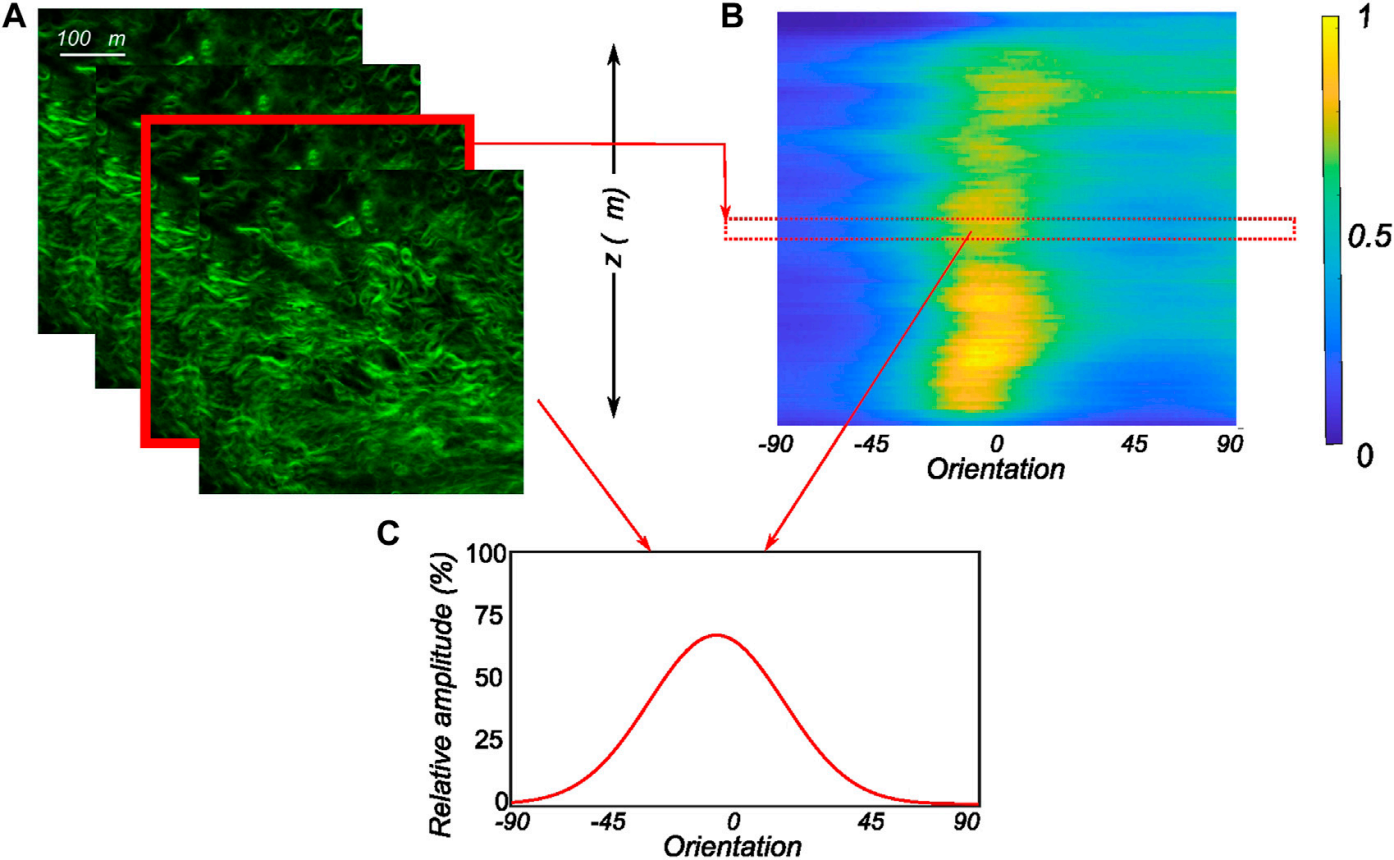
Postprocessing of the obtained MPM image stacks **(A)** The recorded SHG signals through the thickness of the tissue in 2 *μm* intervals with each image in the stack analyzed to extract the planar orientation distribution. **(B)** The computed orientation distributions of all images of the MPM stack as an intensity plot with 1 representing the peak of collagen intensity and 0 representing low collagen intensity. **(C)** The corresponding planar orientation distribution of an individual slice of the MPM stack along with its relative position in the 3D intensity plot.

Here, *a* is the concentration parameter defining the dispersion of the fiber family, and *μ* is the mean orientation angle of the fiber family. Finally, *I*_0_(*a*) is a zero-order Bessel function of type I, which acts as a normalization parameter. The number of images recorded for each sample varied based on the signal-to-noise ratio and the amount of information that could be extracted. As the scan depth increased, the noise dominated the images, which were therefore discarded. The accepted MPM scan images were then analyzed independently to extract the fiber morphology. To get a representative fiber morphology of the entire tissue stack, the average orientation distribution over the entire image stack was computed and used in the modeling process. In addition to that, the relative amplitude of the orientation quantified on all the slices of the entire stack was compiled together to create 3D intensity plots in Matlab as in Cavinato et al. (2017). For this, the obtained relative amplitude values were normalized between 0 and 255 (8-bit image) to produce the color map of the stack’s orientation distributions. An example of a 3D intensity plot and its relation to the planar fiber distribution of a given image slice is shown in Figure 3.

### 2.6 Constitutive model

An anisotropic hyperelastic constitutive model as presented in Holzapfel et al. (2015) is used for describing the mechanical behavior of the human menisci. The deformation gradient *F* is defined in relation to an undeformed reference configuration, that is, assumed to be stress-free. The right Cauchy-Green tensor is then calculated as *C* = *F* ^*T*^ *F*. The modified counterpart of *C* with first invariant *I*_1_ = *tr*(*C*) can be written as $\bar{C}={\bar{F}}^{T}\bar{F}$ with first invariant ${\bar{I}}_{1}=tr\left(\bar{C}\right)$ , where *tr*(•) denotes the trace of (•). A special case of the model presented in Holzapfel et al. (2015) to represent a planar fiber dispersion is used in this study. The strain–energy function *ψ* is decoupled into an isotropic part *ψ*_*iso*_ and an anisotropic (fiber) part *ψ*_*f*_.

$$\psi =\psi_{\mathrm{iso}}+\psi_{f}$$ (3)

The isotropic function is assumed as the neo-Hookean model and *ψ*_*iso*_ is given by

$$\psi_{\mathrm{iso}}=c_{1}\left({\bar{I}}_{1}-3\right)$$ (4)

where *c*_1_ > 0 is a material parameter. The anisotropic part of the strain energy density function is described as follows:

$$\psi_{f}=\frac{k_{1}}{2k_{2}}\left[\exp\left(k_{2}{\bar{E}}^{2}\right)-1\right]$$ (5)

where *k*_1_ is a parameter with the dimensions of stress and *k*_2_ a dimensionless parameter, while

$$\bar{E}=H:\bar{C}-1\;;\;H=\gamma I+\left(1-2\gamma \right)\left(M⊗M\right)$$ (6)

where *I* is the second-order identity tensor, *M* is a unit vector in the direction of the mean orientation of the fiber family, and *γ* is the dispersion parameter of the fiber family. The dispersion parameter is related to the von Mises concentration parameter through the equation $\gamma =0.5-\frac{J_{1}\left(a\right)}{2J_{0}\left(a\right)}$ , where *a* is the concentration parameter defining the dispersion of the fiber family and *J*_0_ and *J*_1_ are modified Bessel function of first kind with order 0 and 1 respectively.

Given the planar biaxial loading, we denote the subscripts *c* and *r* to represent the two in-plane orthogonal directions: circumferential and radial, respectively. The out-of-plane axis is represented by the subscript *z*. The stretch components *λ*_*c*_ and *λ*_*r*_ are computed from the DIC measurements. The condition of incompressibility gives *λ*_*z*_ = $\frac{1}{\lambda_{c}\lambda_{r}}$ . The deformation gradient tensor and right Cauchy-Green tensor can be written in terms of principal stretches as:

$$F=\mathrm{diag}\left[\lambda_{c},\lambda_{r},\lambda_{z}\right]\;;\;C=\mathrm{diag}\left[\lambda_{c}^{2},\lambda_{r}^{2},\lambda_{z}^{2}\right]$$ (7)

The mean fiber orientation unit vector can be written as *M* = *cos*(*μ*)*e*_*c*_ + *sin*(*μ*)*e*_*r*_, where *μ* is the angle between *M* and *e*_*c*_. The unit vectors *e*_*c*_ and *e*_*r*_ are along the circumferential and radial direction, respectively. We define *h* = *FHF* ^*T*^ such that *tr*(*h*) = *tr*(*CH*), whose diagonal components are defined in Eq. 8.

$$\begin{array}{cc} h_{11} & =\left[\gamma +\left(1-2\gamma \right)\cos^{2}\left(\mu \right)\right]\lambda_{c}^{2};\\ h_{22} & =\left[\gamma +\left(1-2\gamma \right)\sin^{2}\left(\mu \right)\right]\lambda_{r}^{2};\\ h_{33} & =0 \end{array}$$ (8)

Hence, the anisotropic components of the Cauchy stress tensor can be written as:

$$\begin{array}{cc} \sigma_{c} & =2k_{1}\left(tr\left(CH\right)-1\right)\exp\left[k_{2}{\left(tr\left(CH\right)-1\right)}^{2}\right]h_{11};\\ \sigma_{r} & =2k_{1}\left(tr\left(CH\right)-1\right)\exp\left[k_{2}{\left(tr\left(CH\right)-1\right)}^{2}\right]h_{22};\\ \sigma_{z} & =0 \end{array}$$ (9)

The isotropic components of the Cauchy stress tensor are

$$\begin{array}{cc} \sigma_{iso_{c}} & =c\left[\lambda_{c}^{2}-\lambda_{z}^{2}\right]\\ \sigma_{iso_{r}} & =c\left[\lambda_{r}^{2}-\lambda_{z}^{2}\right] \end{array}$$ (10)

Together with Eq. 9 and Eq. 10, the non-zero components of the Cauchy stress are

$$\begin{array}{cc} \sigma_{c}^{\mathrm{total}} & =\sigma_{iso_{c}}+\sigma_{c}\\ \sigma_{r}^{\mathrm{total}} & =\sigma_{iso_{r}}+\sigma_{r} \end{array}$$ (11)

where *c* and *r* represent the circumferential and radial components respectively. It is to be noted that the stress components in Eq. 11 are derived by enforcing an incompressibility constraint, meaning the isotropic component is not a direct derivative of Eq. 4. The isotropic material parameter is presumed to account for the contributions of the ground matrix and elastin. Based on MPM investigations, a single fiber family was chosen for the layers of the menisci, whose stress components are described by Eq. 9 and Eq. 10.

### 2.7 Inverse material parameter identification

Quantified fiber morphology derived from MPM image stacks reduced the number of parameters to be identified. A cost function representative of the standard error of estimate (SEOE) (Peddada et al., 2005), based on the experimental and analytical stress-stretch responses, was used in the optimization process. The structural parameters: mean fiber orientation and fiber dispersion were identified from the SHG image stacks. The material mechanical parameters (*c*_1_, *k*_1_, *k*_2_) were inversely identified in the parameter optimization process. Constraints were imposed based on the parameters in order to ensure a uniform sampling of the initial population. Multiple runs of the optimization algorithm as well as the second-order derivatives at the optimized point aimed at avoiding the identified parameter set that did not correspond to local minima. The identified parameter set obtained by minimizing the cost function was accepted as the optimal value.

$$f=\sqrt{\frac{\overset{n}{\underset{i=1}{\sum }}{\left(\sigma_{\exp}\left(i\right)-\sigma_{\mathrm{Analytical}}\left(i\right)\right)}^{2}}{n}}$$ (12)

In the above equation, *n* is the number of data points considered for each sample, following a spatial discretization of stretch (*n* > 20). *σ*_*exp*_ and *σ*_*analytical*_ are the experimental and analytical stress values at each point on the discretized stretch domain.

## 3 Results

### 3.1 Biaxial mechanical response of human menisci

**FIGURE 4:**
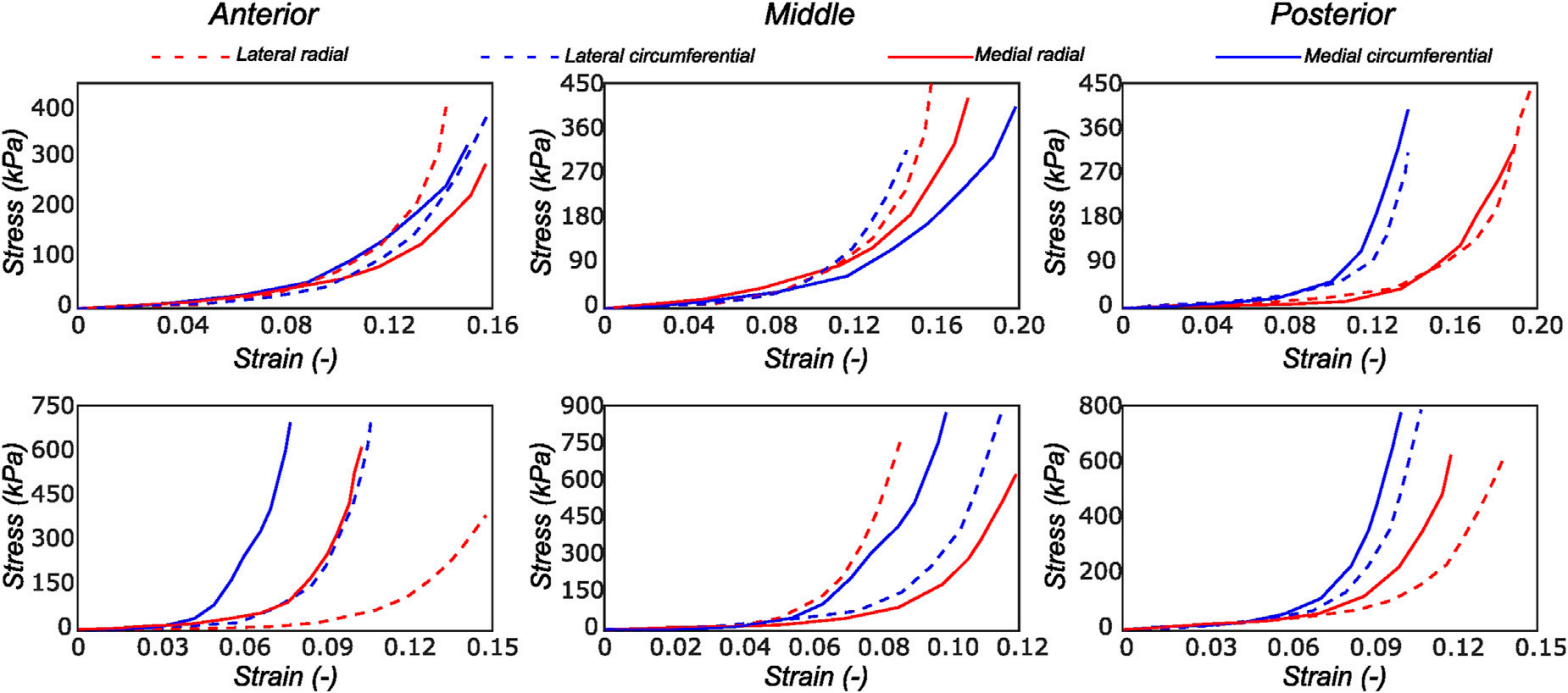
Computed Cauchy stress-strain curves for the meniscus samples (LM1 and MM1) obtained from the biaxial testing protocol. The top and bottom rows of subplots represent the samples from the top and bottom samples, respectively. Column-wise, they represent the anterior, mid-body, and posterior regions.

We observed the typical non-linear, anisotropic membrane stress-stretch response of the meniscal layers under all loading protocols. For the top samples, the radial and the circumferential directions displayed similar extensibility. The coupling between the two tissue directions was also evidenced in the non-equibiaxial tension protocols. For example, under circumferentially dominant tension protocols, radial stress decreased while circumferential stress increased. The mechanical response varied significantly with the region and layer of the sample. Figure 4 shows the delineated mechanical response of samples tested from a lateral and a medial meniscus, respectively. Comparing the two menisci as a whole, the medial menisci were found to be stiffer in the circumferential direction (upto 14%), while the radial direction was not significantly different among the two menisci (the difference is ≤3.4% across all the biaxial loading protocols).

### 3.2 Quantification of collagen fiber morphology

The thickness of human menisci can go up to 6 mm. Although imaging of the collagen fiber architecture through the whole thickness is extremely challenging, the optical clearing technique used in this study enabled us to image individual layers up to 500 *μ*m.

#### 3.2.1 Lateral menisci

**FIGURE 5:**
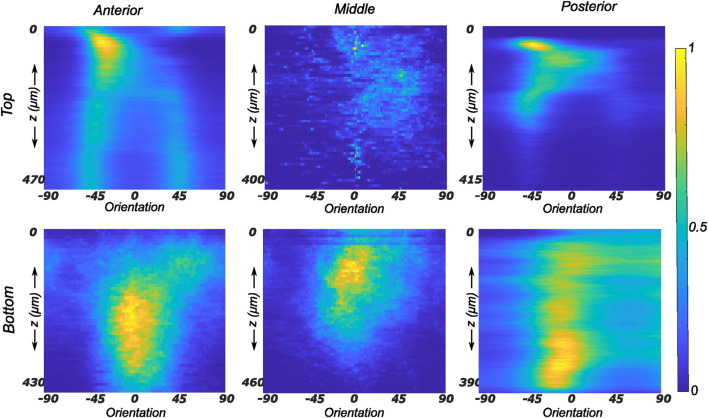
Depth profile of collagen fiber intensity of samples obtained from the lateral meniscus. The images recorded on the entire stack were analyzed individually and compiled together in Matlab to produce an intensity plot representative of the transmural variation in collagen fiber morphology. The top and bottom rows of subplots represent the top and bottom samples, respectively. Column-wise, they represent the anterior, mid-body, and posterior regions.

**FIGURE 6:**
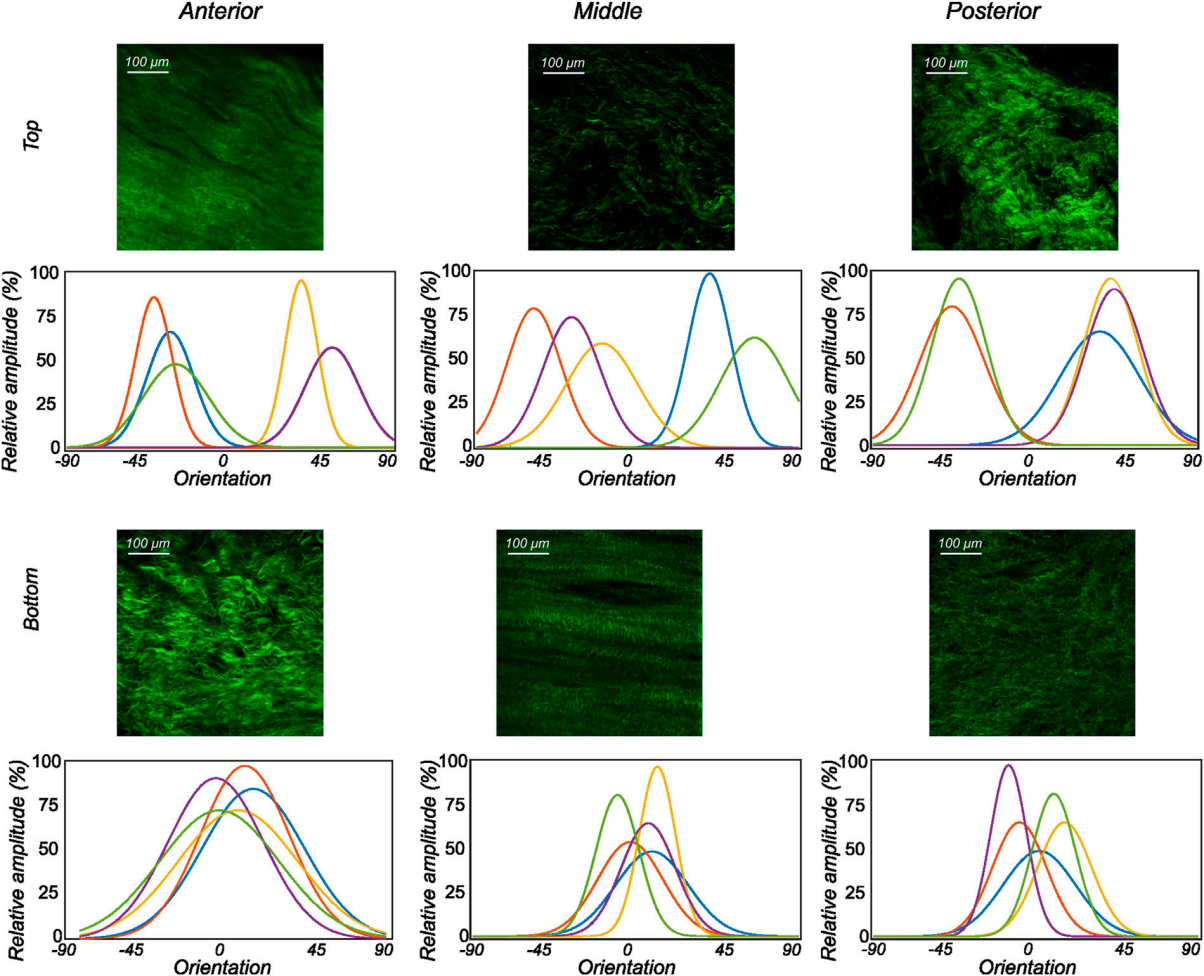
The orientation distribution computed on each image slice of individual image stacks was averaged through the thickness to obtain the representative collagen orientation distribution of the sample. Here, the computed orientation distribution for samples excised from lateral menisci is shown. The top and bottom profiles represent the samples from the top and bottom samples, respectively. Column-wise, they represent the anterior, mid-body, and posterior regions.

The collagen fiber morphology through the imaged thickness for samples obtained from different regions of a representative lateral meniscus is shown in Figure 5 as a contour plot. The color bar depicts the relative intensity of the fibers along a given orientation (*x*-axis) and a given depth (*y*-axis). As illustrated in Figure 5, all samples show rich collagen content with their orientation evolving through the thickness of the top layer. The distinctive characteristic of the top samples was the presence of randomly organized fibers, with the mean fiber orientation varying with the anatomical regions as shown in the top row subplots of Figure 5. The mean fiber orientation (*μ* in Table 1) of samples from the top layer was −33.2 ± 12.8° in the anterior region, to −40.1 ± 10.2° in the mid-body, and 36.7 ± 15.7° in the posterior region. On the contrary, the samples from the bottom layer were characterized by densely packed collagen fibers predominantly oriented in the circumferential direction. This was observed for all thicknesses and for the three anatomical regions (anterior, mid-body, and posterior), as illustrated in the bottom row profiles of Figure 5. The mean fiber orientation was 4.7° in the anterior region, −2.4° in the mid-body, and −8.5° in the posterior region. Within each region, the variation in computed dispersion of collagen fiber orientations was ≤5% through the thickness. Conversely, it was seen that the dispersion of fibers was higher in the top samples with an average standard deviation of 12.7° as compared to 8.8° in the bottom layer samples. Figure 6 shows the average collagen fiber orientation distribution in all the imaged samples. It can be seen that while the aggregate orientation distribution varies significantly in the top samples, it is insignificant in the bottom samples. The corresponding von Mises distribution parameters as well as the inversely identified anisotropic material parameters for five lateral menisci are presented in Table 1.

**TABLE 1:** Collagen morphology and tissue material parameters for top and bottom samples from lateral menisci. LM denotes lateral meniscus.

Sample	Region	*μ* (deg)	*a*	*c*_1_ (kPa)	*k*_1_ (kPa)	*k*_2_ (−)	*γ* (−)	*r* ^2^
Top samples								
LM1	Ant	−33.2	2.38	12.7	27.4	7.2	0.12	0.92
	Mid	40.1	2.77	8.5	16.1	5.8	0.11	0.94
	Pos	36.4	0.85	13.2	28.2	12.2	0.32	0.91
LM2	Ant	−42	3.36	7.2	18.2	9.5	0.09	0.94
	Mid	−58.7	1.87	3.9	20.4	8.1	0.19	0.93
	Pos	−46.4	1.31	5.2	27.6	8.4	0.25	0.93
LM3	Ant	39.9	4.23	3.8	13.8	11.5	0.07	0.91
	Mid	−20.4	1.08	5.7	21.4	6.8	0.28	0.93
	Pos	42.1	1.82	3.2	19.7	13.4	0.18	0.92
LM4	Ant	56.8	1.81	14.8	28.4	11.2	0.18	0.93
	Mid	−37.4	1.56	21.4	21.6	5.8	0.20	0.92
	Pos	44.2	1.56	17.1	20.7	10.2	0.19	0.94
LM5	Ant	−30.4	1.24	13.2	25.2	9.4	0.24	0.95
	Mid	65.1	1.12	8.8	23.5	7.6	0.27	0.95
	Pos	−42.4	1.69	5.7	28.1	8.1	0.16	0.93
Bottom samples								
LM1	Ant	12.2	1.07	6.4	22.8	3.9	0.28	0.92
	Mid	9.1	1.07	10.5	36.2	11.4	0.28	0.94
	Pos	3.4	1.08	7.2	28.2	6.8	0.27	0.91
LM2	Ant	15.1	1.56	6.6	18.2	8.6	0.19	0.94
	Mid	−3.7	3.67	2.4	23.6	7.4	0.25	0.93
	Pos	−8.4	1.89	3.2	31.4	5.4	0.17	0.93
LM3	Ant	3.9	0.63	5.1	24.3	8.2	0.37	0.91
	Mid	−12.4	1.87	7.7	21.4	12.2	0.08	0.93
	Pos	13.1	1.81	3.8	39.7	6.4	0.15	0.92
LM4	Ant	−16.8	1.22	3.4	21.4	10.4	0.23	0.93
	Mid	7.4	2.96	12.1	18.6	8.3	0.17	0.92
	Pos	14.2	3.72	10.5	15.7	11.2	0.09	0.94
LM5	Ant	−8.4	0.68	3.8	22.4	8.7	0.34	0.95
	Mid	−9.1	1.12	5.1	17.4	9.4	0.11	0.95
	Pos	10.4	2.69	4.6	18.1	12.3	0.11	0.93

#### 3.2.2 Medial menisci

**FIGURE 7:**
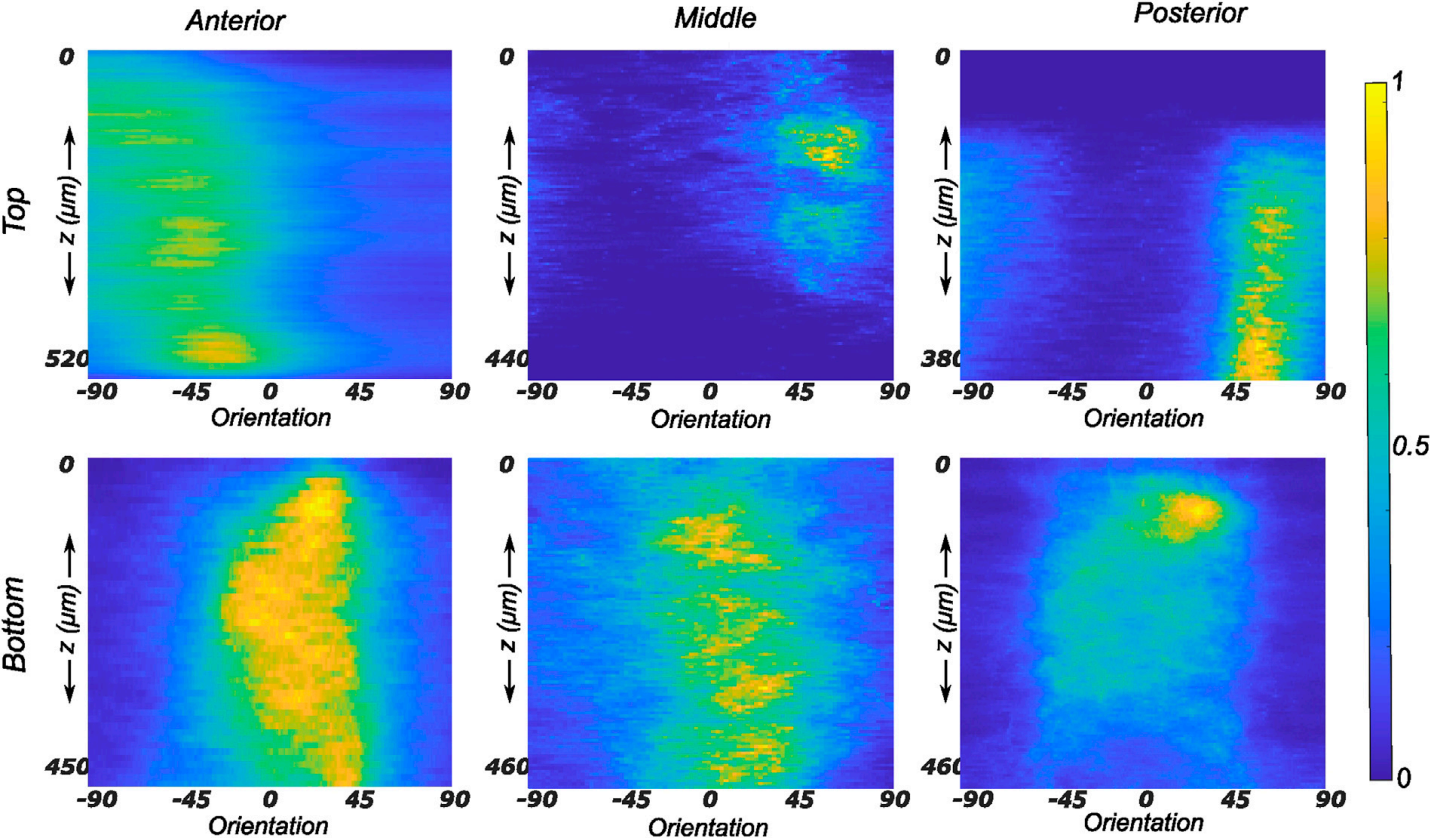
Depth profile of collagen fiber intensity of samples obtained from the medial meniscus. The images recorded on the entire stack were analyzed individually and compiled together in Matlab to produce an intensity plot representative of the transmural variation in collagen fiber morphology. The top and bottom rows of subplots represent the top and bottom samples, respectively. Column-wise, they represent the anterior, mid-body, and posterior regions.

**FIGURE 8:**
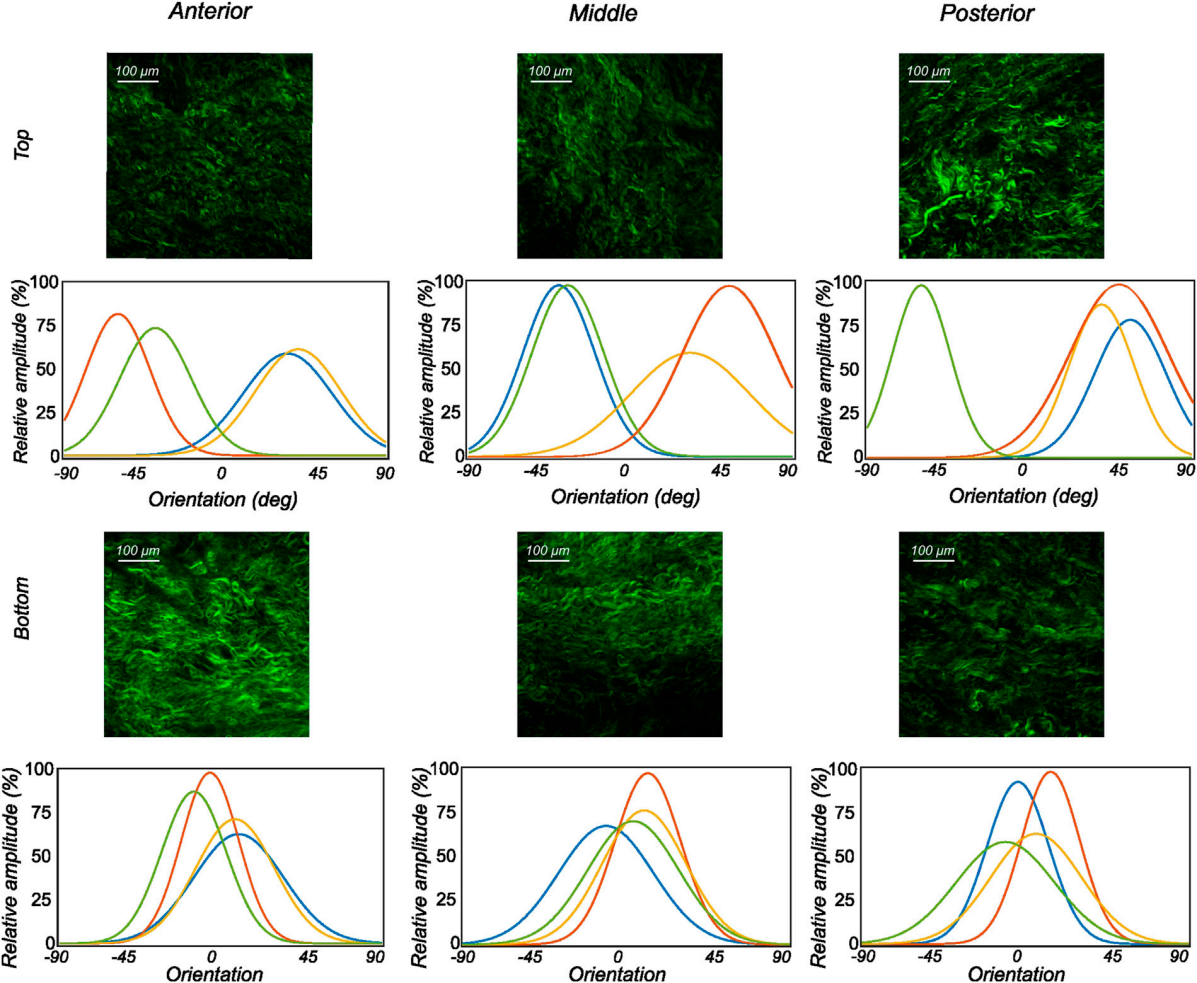
The orientation distribution computed on each image slice of individual image stacks was averaged through the thickness to obtain the representative collagen orientation distribution of the sample. Here, the computed orientation distribution for samples excised from medial menisci is shown. The top and bottom profiles represent the samples from the top and bottom samples, respectively. Column-wise, they represent the anterior, mid-body, and posterior regions.

The fiber morphology in the medial menisci showed similar characteristics to the lateral menisci. The fibers in the top samples were sparsely arranged, and the preferred orientation varied between different regions and among different menisci. For instance, as shown in the top row profiles of Figure 7, the mean fiber orientation (*μ* in Table 2) of samples from the top layer varied from −60.8 ± 13.6° in the anterior region to 55.6 ± 17.1° in the middle, to 49.4 ± 20.2° in the posterior region. Within the bottom layer, in terms of their preferred orientation, the fibers were aligned close to the circumferential direction (bottom row profiles in Figure 7). The mean fiber orientation was computed to be 9.4° in the anterior region, 9.2° in the mid-body, and 9.1° in the posterior region (absolute value away from 0). The dispersion of fibers in both layers was observed to be similar, contrary to lateral menisci. The medial menisci were found to have a significantly higher dispersion of fibers within the top and bottom samples than the lateral menisci (18.3 vs. 11.6°). In contrast to the lateral menisci, the fiber morphology of bottom samples showed a higher degree of variation among the medial menisci, as shown in Figure 8. The corresponding von Mises distribution parameters as well as the inversely identified anisotropic material parameters for four medial menisci are tabulated in Table 2.

**TABLE 2:** Collagen morphology and tissue material parameters for top and bottom samples from medial menisci. MM denotes medial meniscus.

Sample	Region	*μ* (deg)	*a*	*c*_1_ (kPa)	*k*_1_ (kPa)	*k*_2_ (−)	*γ* (−)	*r* ^2^
Top samples								
MM1	Ant	35.4	0.64	31.4	32.7	7.8	0.35	0.96
	Mid	−40.7	0.98	24.5	37.6	6.4	0.29	0.94
	Pos	56.2	0.93	23.7	30.1	6.8	0.31	0.95
MM2	Ant	−60.8	1.26	18.6	40.7	9.5	0.24	0.93
	Mid	55.6	0.57	13.7	32.1	12.2	0.36	0.92
	Pos	49.4	0.54	17.1	24.9	8.4	0.38	0.92
MM3	Ant	41.3	0.69	14.8	36.7	11.2	0.33	0.91
	Mid	53.8	0.40	11.3	28.2	8.8	0.40	0.94
	Pos	40.9	1.28	20.2	30.4	9.6	0.24	0.92
MM4	Ant	−39.1	1.02	11.5	29.5	12.4	0.28	0.92
	Mid	−35.7	1.04	15.7	26.7	8.4	0.28	0.91
	Pos	−60.3	1.57	8.4	33.4	10.1	0.21	0.93
Bottom samples								
MM1	Ant	10.4	0.64	9.4	41.2	7.4	0.40	0.96
	Mid	−11.7	0.59	12.5	17.6	11.6	0.37	0.94
	Pos	−4.2	1.43	13.7	20.3	9.4	0.21	0.95
MM2	Ant	−6.8	1.66	8.2	25.7	11.6	0.20	0.93
	Mid	12.6	1.32	11.6	22.6	8.1	0.26	0.92
	Pos	14.4	1.54	7.4	18.7	10.4	0.18	0.92
MM3	Ant	8.3	0.89	4.3	22.5	13.9	0.31	0.91
	Mid	10.8	0.76	1.9	18.2	7.3	0.29	0.94
	Pos	6.9	0.78	2.7	31.1	9.7	0.34	0.92
MM4	Ant	−14.1	1.22	5.8	28.3	13.4	0.24	0.92
	Mid	4.7	0.69	10.7	14.9	10.6	0.36	0.91
	Pos	−11.3	0.57	8.1	23.4	13.1	0.41	0.93

### 3.3 Constitutive modeling

**FIGURE 9:**
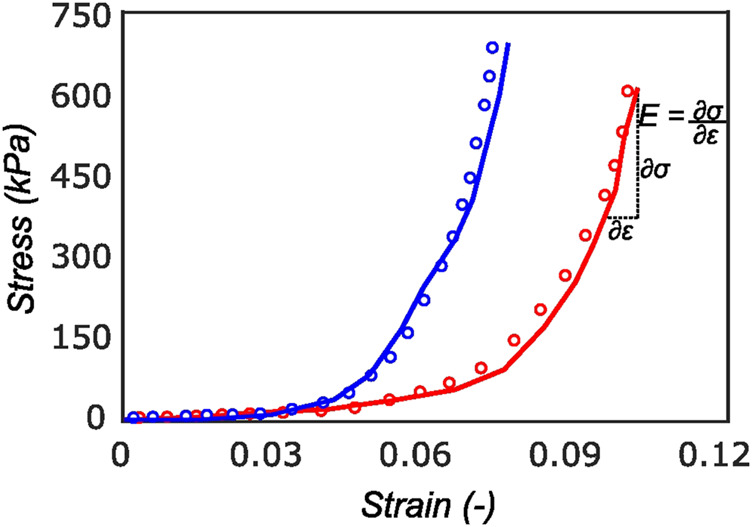
Cauchy stress *versus* strain for a sample in the radial (red curve) and circumferential direction (blue curve). The experimental data (solid curve) is well represented by the model (dotted curve) with *r* ^2^ of 0.94. On the final loading regime of the red curve, the computation of linear elastic modulus is illustrated. Most of the tissue stiffness in this loading region is assumed to be contributed by fibers.

The proposed constitutive model in Section 2.6 takes into account the mean fiber morphology in the tested tissue sample to better describe the anisotropy of the meniscal tissue. Figure 9 illustrates the predictive capability of the constitutive model on a single sample, and it is evident that the model is in good agreement with the experiment data. The constitutive model parameters for the top and bottom samples from the anterior, mid-body, and posterior regions of each lateral and medial meniscus are tabulated in Table 1 and Table 2, respectively. The derived von Mises distribution parameters are included in columns 5, 6, and 7. In almost all samples examined, a single fiber family was deemed sufficient to satisfactorily represent the collagen fiber morphology within the tissue. However, the anterior segment from lateral meniscus 2 (LM2) was found to have two perpendicular mean fiber orientations across its thickness. To simplify, the fiber orientations were averaged into a single vector, thereby approximating it with a single fiber family. Nonetheless, a single representative fiber family in a constitutive tissue model could adequately capture the mechanical behavior of the sample. The constitutive model predicts the mechanical behavior of the tissue samples with good agreement, and an average *r* ^2^-value is 0.94. Overall, the isotropic material constant for the extracellular matrix was found to be relatively small (10.4 ± 5.8 kPa). The anatomical specificity, including the region and depth variability, is obvious from Table 1 and Table 2.

## 4 Discussion

In this paper, the structure-based mechanics of human menisci are quantified using an anisotropic hyperelastic material model. The collagen fiber morphology is examined within five lateral menisci and four medial menisci at three different anatomic regions and two layers through the depth of the tissue. SHG imaging combined with planar biaxial testing permits a high degree of coupling between the material’s anisotropic mechanical response and the underlying collagen fiber morphology. To the best of our knowledge, this is the first study to explore the collagen fiber morphology throughout the human meniscal tissue and incorporate it into the tissue’s mechanical response.

We found that using the SeeDB (Ke et al., 2013) optical clearing protocol, it is possible not only to visualize, but also to quantitatively analyze the local microstructural organization of collagen network in anterior, mid-body, and posterior regions of the human menisci in 3D. Each sample was imaged to a depth of up to 500 *μm* through the thickness. An FFT (Fast Fourier Transform)-based automatic algorithm was used to quantify the distribution of collagen fibers. The automated process ensures high reproducibility but poses some challenges. The Fourier transform assumes a periodic signal, so a window should be applied to the images before the Fourier transform. In the current study, a tapered cosine window is used to minimize the loss of information at the edges of each image (layer). The results of the local microstructural orientation analysis indicate that there are substantial differences in collagen organization in the top samples. The mean orientation was often computed to be around the diagonal direction (±45°) with high dispersion. Conversely, the samples from the bottom layer showed consistent collagen fiber organization close to the circumferential direction with varying dispersion. Evidently, the majority of collagen found in the bottom layers of the human menisci was organized into circumferential bundles with some randomly oriented fibers.

This study has refined the understanding of collagen fiber structure in the human meniscus. Several imaging techniques have been previously utilized to characterize the collagen fiber morphology in meniscus tissue; including polarized light microscopy (PLM) (Kiraly et al., 1997; Danso et al., 2017), second harmonic generation (SHG) via multiphoton microscopy (Brown et al., 2014; Vetri et al., 2019), small-angle x-ray diffraction (Aspden et al., 1985; Moger et al., 2007), scanning electron microscopy (Zhu et al., 2021), and *μ*CT (Karjalainen et al., 2021). Apart from *μ*CT examinations, all other studies were limited to an imaging depth of 100 *μm* in human meniscus tissue. The *μ*CT investigation covers a volume of interest of 1800 *μm* ^3^. At this point, direct comparisons could be made. The X-ray diffraction conducted on two human samples (Aspden et al., 1985) showed that the top superficial layer is dominated by a mixture of radial and randomly oriented collagen fibers, extending up to a depth of 100 *μm*. Beyond this depth, they report circumferentially oriented fiber bundles within the bulk of the tissue. The SHG imaging conducted in Vetri et al. (2019) describes in detail the collagen fiber architecture in both circumferential and radially oriented samples, with the transmural imaging scan depth limited to 60 *μm* as compared to 500 *μm* in the current study. However, their results from the circumferential sections of a human lateral meniscus are in agreement with our observations, where the mean orientation of collagen fibers is 9.5 ± 3.2° compared to their 11°. While their study was extensively focused on explaining the micro-scale and nano-scale architecture of collagen fibers in the human menisci, we are more interested in combining the mean collagen morphology with the tissue’s mechanical response.

Due to the non-homogeneous composition and the anisotropic mechanical properties of the meniscus, the local stress–strain response varies greatly with location. Therefore, the experimental testing apparatus should encompass tensile, compressive, and shear stresses and strains. Confined compression tests conducted on the tissue (Proctor et al., 1989; Joshi et al., 1995) are important to better understand the bi-phasic environment of the meniscus. Biomechanical parameters such as compressive stiffness, tissue permeability, and aggregate water content could be derived from such testing protocols, which are all complex material modeling and simulations. Dynamic shear testing (Anderson et al., 1991) reveals the aggregate region-specific shear modulus of the tissue given that shear loading is one of the primary mechanisms for meniscus tear (Siegel, 1988). However, considering that the meniscus is rich in collagen fibers which do not contribute to compressive load bearing, tensile testing is important for understanding their role in the maintenance, degeneration, and repair of the meniscus. The majority of tensile testing was limited to uniaxial testing protocols (Proctor et al., 1989; Tissakht and Ahmed, 1995; Goertzen et al., 1997), which limits the understanding of material in-homogeneity and anisotropy. In the current study, we quantify the biaxial tensile material properties of nine human menisci with respect to regional and depth-wise variations. This is a necessary improvement on existing literature as the tissue undergoes *in-vivo* loading in multiple directions simultaneously (Walker and Erkiuan, 1975).

Based on our region- and layer-specific studies of the human menisci, we can conclude that its mechanical response is highly anisotropic. Therefore, the constitutive model should be complex enough to reflex this behavior. Early studies were limited to the premise of linear elasticity (Tissakht and Ahmed, 1995), but based on the results of experimental testing, transversely isotropic (Wheatley et al., 2015) and anisotropic hyperelastic material models (Abraham et al., 2011) have been developed (tension, indentation, shear, *etc.*). The extent of non-linear anisotropic hyperelastic material models used for describing the mechanics of meniscal tissue is either limited to phenomenological descriptions (Haemer et al., 2012) or structure-based models that take into account the morphology of collagen fibers in the tissue (Freutel et al., 2015; Seyfi et al., 2018; Abraham et al., 2011). However, the studies reported in Freutel et al. (2015); Seyfi et al. (2018) are based on inverse finite element modeling using indentation testing data, which is not sufficient to capture the complex anisotropic behavior of the tissue. Moreover, they did not conduct any histological imaging but identified collagen fiber morphology parameters inversely, which can be misleading. On the other hand, the study on the bovine menisci reported in Abraham et al. (2011) utilizes previously reported micro-structural imaging data (Hutton, 1993) and assumed two perpendicular fiber families in their modeling process. While this is well-founded, the consideration of collagen fiber dispersion could further enhance the material modeling. In the current study, we incorporated region-specific and layer-specific collagen fiber morphology in the constitutive model to better explain the structure-function relationship in the tissue’s mechanical response. We included fiber dispersion details in addition to the fiber orientation; both of these material parameters are indicators of collagen fiber morphology and were derived from SHG imaging. This is intuitive since the amount of reorganization of fibers during loading influences the mechanical response in different directions, which cannot be expressed by just considering the mean fiber orientation.

The estimated values of *k*_1_ and *k*_2_ in Table 1 and Table 2 represent the fiber properties, which are stress-like and dimensionless parameters, respectively. The average values of these parameters for the lateral meniscus are 23.3 ± 5.9 kPa and 8.8 ± 2.3; and for the medial meniscus, they are 27.8 ± 7.2 kPa and 9.9 ± 2.1. This indicates the anatomical specificity of the human meniscus, with the medial meniscus being stiffer than the lateral meniscus. The average fiber properties of samples from six locations tabulated in Table 3 confirm the width- and depth-wise difference in material properties. This variation is stronger in the medial menisci than in the lateral menisci. Among the samples from the top layer in the medial menisci, the anterior region showed higher values of fiber properties (*k*_1_ and *k*_2_ are 34.9 ± 4.8 kPa and 10.2 ± 2.0, respectively), while in the lateral menisci, the posterior region displayed higher values (*k*_1_ and *k*_2_ are 24.8 ± 4.2 kPa and 10.4 ± 2.3, respectively). This pattern was also observed in the bottom layer sample. When anatomical specificity was compared along with region- and layer-specificity, the top layer from the anterior horn of the medial menisci had the marginally highest stiffness with average *k*_1_ and *k*_2_ values (34.9 ± 4.8 kPa and 10.2 ± 2.0).

**TABLE 3:** The average values of *k*_1_ and *k*_2_ for samples from different locations of the lateral and medial menisci.

Sample location	*k*_1_ (*kPa*)		*k*_2_	
	Lateral meniscus	Medial meniscus	Lateral meniscus	Medial meniscus
Anterior top	22.60 ± 6.32	34.90 ± 4.86	9.76 ± 2.80	10.22 ± 2.00
Middle top	20.60 ± 2.57	31.15 ± 4.68	6.82 ± 1.04	8.95 ± 2.40
Posterior top	24.86 ± 4.27	29.70 ± 3.52	10.46 ± 2.32	8.72 ± 1.46
Anterior bottom	21.82 ± 2.27	29.42 ± 8.20	7.96 ± 2.42	11.57 ± 2.95
Middle bottom	23.44 ± 7.53	18.32 ± 3.19	9.74 ± 2.02	9.40 ± 2.03
Posterior bottom	26.62 ± 9.85	23.37 ± 5.50	8.42 ± 3.10	10.65 ± 1.68

**FIGURE 10:**
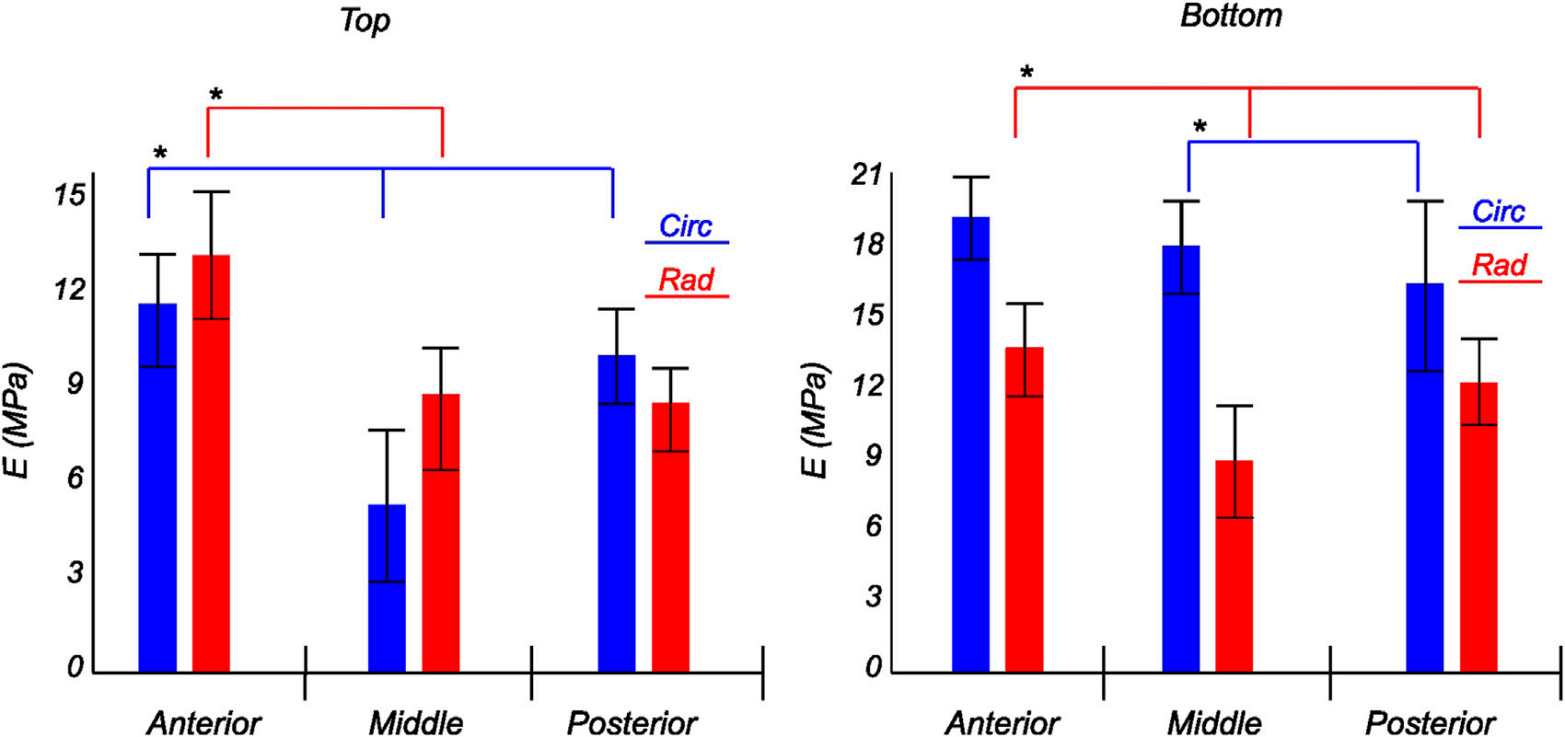
Computed elastic modulus of a human lateral meniscus in the final loading regime: top layer (left), and bottom layer (right). The anisotropic nature of the material is shown with the circumferential stiffness represented in blue and the radial stiffness represented in red. Within each layer, the anterior, middle, and posterior regions are presented to see the regional variations. * indicates statistical significance from a one-way ANOVA test with *p*-value < 0.05.

**FIGURE 11:**
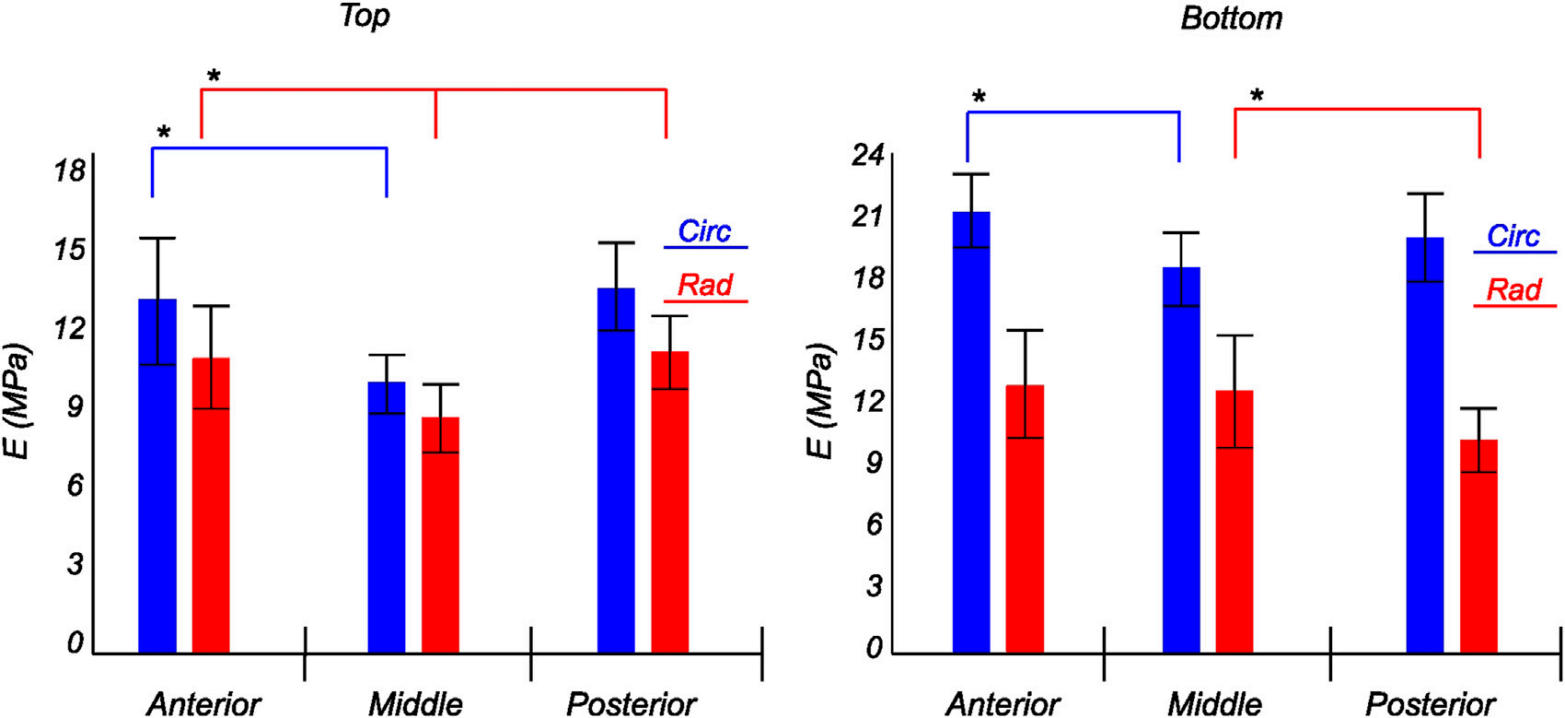
Computed elastic modulus of a human medial meniscus in the final loading regime: top layer (left), and bottom layer (right). The anisotropic nature of the material is shown with the circumferential stiffness represented in blue and the radial stiffness represented in red. Within each layer, the anterior, middle, and posterior regions are presented to see the regional variations. * indicates statistical significance from a one-way ANOVA test with *p*-value < 0.05.

**FIGURE 12:**
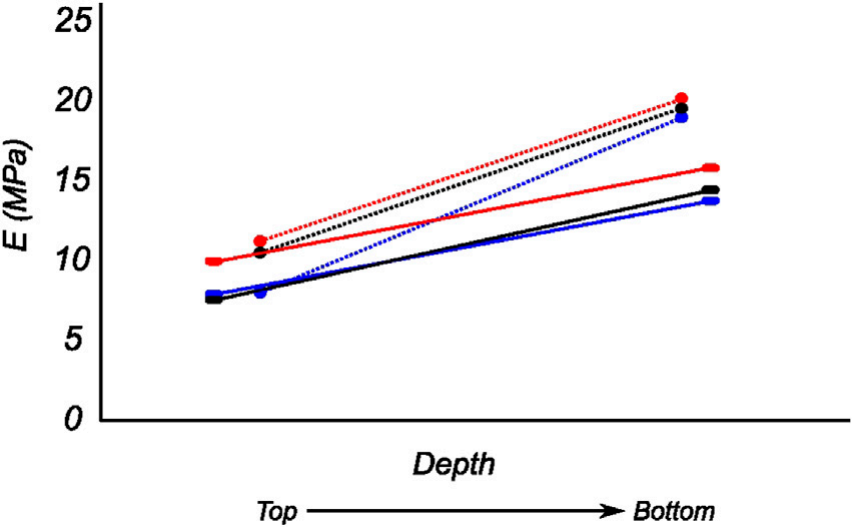
Computed biaxial elastic modulus of all meniscus samples showing regional and depth-wise variation. The circumferential and radial moduli are shown as dotted and solid lines respectively. Regions anterior (red), middle (blue), and posterior (black) are presented here.

The tangent modulus in the final loading regime (see Figure 9) has been computed to take into account the stiffness of tissue post collagen fiber engagement. The overall pooled results show that the circumferential direction was significantly stiffer than the radial direction. This was expected given the presence of primarily arranged circumferential fiber bundles in all the bottom-layer samples and a mixture of circumferential and near-diagonally oriented fiber bundles in top samples. This is in accordance with previously conducted tensile tests (Tissakht and Ahmed, 1995; Kahlon et al., 2015). Further assessment of the regional and layer-specific results revealed substantially higher circumferential elastic moduli in the anterior regions of both menisci (lateral and medial) within bottom-layered samples (see Figure 10 and Figure 11). A similar trend was not observed in the top samples, with both circumferential and radial moduli being comparable to each other. Within both the top and bottom samples, the medial menisci displayed a slightly higher modulus compared to the lateral menisci. An interesting comparison to note here is the study by Proctor et al. (1989), where uniaxial tension tests in the superficial layer revealed a near isotropic behavior with a mean elastic modulus of 59.4 *MPa*. The reported decrease in radial elastic modulus with the depth of the tissue is not seen in our observations, as shown in Figure 12. This could possibly be due to two reasons: firstly, regional sectioning data was not mentioned in their study, which could create some ambiguity; secondly, fiber kinematics are not well represented in uniaxial tension, which often leads to an underestimation of elastic modulus in directions away from principle fiber orientations. Our findings demonstrate that both the circumferential and radial moduli increase with depth, while the circumferential modulus showed more increment. A one-way ANOVA test was used to evaluate the statistical significance between different groups of elastic moduli in lateral and medial menisci (anterior, middle, and posterior regions; top and bottom layers), as shown in Figures 10, 11. Within each layer, all three regions were compared independently in circumferential and radial directions. A *p* − *value* < 0.05 was considered to be representative of statistical significance. As shown in Figure 10, the elastic moduli in the top layer were found to vary significantly between regions in the circumferential direction, while the elastic moduli in radial direction were found to vary significantly between anterior and middle regions. Similarly, in the bottom layer, elastic moduli were found to vary significantly between regions in the radial direction, while the elastic moduli in circumferential direction were found to vary significantly between middle and posterior regions. With reference to Figure 11, the radial elastic moduli were found to be significantly different between all regions in the top layer, while significance was only observed between anterior and middle regions for circumferential elastic moduli. Within the bottom layer, statistical significance was observed between anterior and middle, middle and posterior regions for circumferential and radial elastic moduli respectively. However, it is to be noted that the sample size utilized to conduct this analysis is low (*n* = 4/5) and should be updated as and when more samples are tested. Moreover, the reported range of elastic modulus in our study (∼5−22MPa) is in good comparison with biaxial tension tests conducted on porcine menisci (Kahlon et al., 2015) (∼1−15MPa) . Larger variations of elastic properties (∼35−190MPa) have also been observed in uniaxial testing of porcine menisci (Maritz et al., 2021). These differences may arise due to differences in the properties of the porcine and human meniscus, which may also result from the differences in sample preparation techniques and loading rate.

Our study has the following limitations. The samples used in our study represented a geriatric group because of the difficulties in obtaining younger cadaveric subjects. Even if we used cadavers with no history of arthritis and discarded the ones with visible tears, we cannot guarantee the non-degeneracy of the tissue as it is from elderly people. Using the model herein, we could model both young and old tissue, but it requires sample studies from donors of different age groups (Haskett et al., 2010). Therefore, against the background of patient-specificity based on demography, this study is limited to the older population. Further, we assumed the outcomes of *in vitro* and *in vivo* studies do not contradict much as we used biaxial testing which can better replicate the complex *in vivo* loading conditions (Jacobs et al., 2013; Kahlon et al., 2015). Even though the previous studies reported three distinct layers (Petersen and Tillmann, 1998) such as a superficial, lamellar, and central main layer, our study discards the individual characteristics of superficial and lamellar layers. The superficial layer thickness is approximately 150–200 *μ*m, and the depth of the layer decreases to 20–30 *μ*m towards the internal circumference (Petersen and Tillmann, 1998). Therefore, obtaining samples from this layer by manual slicing was not practicable. Hence, we collected the *top samples* in each region from an approximate zone that combined the superficial and lamellar layers to circumvent the challenge of cutting samples from this paper-thin layer while still including the biomechanical aspects of both layers in our model. However, we paid special attention to extracting the *bottom sample* from the central main layer by removing other layers from the inferior surface of the meniscus. A consistent, repeatable tissue slicing protocol for irregularly shaped tissues such as the meniscus is presented in Bonomo et al. (2020). As an extension to the current study, the protocol could be adopted to improve the sample preparation protocol. For this study, however, since the excised tissue samples do not have a constant thickness, the principal stresses were assumed to be uniform through the thickness, with minimal alterations.

The field of view of SHG imaging (465 × 465 *μm*) is too small compared to the sample size used in biaxial testing. Even though the optical clearing procedure greatly increased the depth of the image stacks, the whole thickness of the tissue was still not captured. Therefore, further improvement in clearing and imaging protocols could enrich our current findings. Along with regulating elastic recoil, elastin fibers also contribute to the load-bearing properties of the tissue. More information may be gained from their structural organization, which was not considered in this study. Although the samples used in the biaxial investigation had a non-uniform thickness that was brought about by the manual slicing of the tissue, our study assumed a uniform stress distribution through the thickness during the post-processing of test data. The constitutive model utilized here only considers the morphology of collagen fibers, disregarding the density of collagen, fiber waviness, and the number of crosslinks. All of these are important factors that control the mechanics of the tissue at the microscale. However, quantifying these parameters is beyond the scope of the current study. The model further assumes affine fiber kinematics, which has been shown not to be the case for soft tissues with a dense network of collagen fibers (Krasny et al., 2017). Moreover, despite the fact that the meniscus tissue is also known to exhibit poroelastic and viscoelastic behavior (Myers et al., 2018; Bulle et al., 2021; Maritz et al., 2021), the current study primarily seeks to advance our understanding of the quasi-static mechanics of the tissue.

The chemical fixation and microscopy protocol is applied to the samples after they undergo biaxial testing. Possible alterations could have been induced to the collagen fiber morphology due to this. The amount of change, if any, has not been quantified in this study. Further, the main advantage of using MPM for imaging collagen is its non-destructive, non-invasive nature. The optical clearing agent SeeDB highly limits the amount of tissue shrinkage in comparison to other clearing methods (Azaripour et al., 2016), while greatly improving the scan depth. However, any changes induced to the collagen morphology are not known/reported at this moment.

## 5 Conclusion

In this study, the collagen structure of meniscal tissue regions was quantified through the thickness with high transmural resolution. The majority of collagen fibers in human menisci are circumferential in nature. The mean fiber angle and collagen dispersion were found to vary across the thickness and within the anterior, mid-body, and posterior regions. Additionally, planar biaxial mechanical tests were performed to characterize the mechanical behavior of meniscal tissue within the regions. Biaxial test results confirmed that the menisci have a significantly higher tensile strength in the circumferential direction than the transverse direction, which explains the injury mechanisms in commonly occurring meniscal tears such as the horizontal flap tear, the vertical longitudinal tear, and the bucket-handle tear (Patel et al., 2021). The biaxial results further affirmed the region- and layer-specific variations in material properties, which emphasize the highly anisotropic nature of the tissue. Furthermore, it was found that the tissue matrix is compliant and that collagen is the main load carrier. The medial menisci exhibited a slightly higher modulus than the lateral menisci, which may be attributed to its stiffer connections with the knee joint capsule and the medial collateral ligament. Hence, the mechanical and morphological observations in our study agree with the clinical findings. Despite the variation in collagen morphology, a constitutive tissue model with a single representative fiber family adequately captured the mechanical behavior of the meniscal tissue. This material model is based on a strain-energy function that accommodates both the mechanical and structural features of the menisci.

The presented constitutive model parameter is extremely important in order to understand the macroscopic behavior of the human menisci and for developing a reality-based model for medical simulators to facilitate adequate training. Knee arthroscopy simulators based on our model could provide realistic tactile feedback during the meniscal probing. The region- and layer-specific material data enables realistic tissue manipulation with respect to applied probing force. Moreover, the slightest variation in tissue behavior during different probing tasks, such as push and continuous run on the superior meniscal surface and pull on the inferior meniscal surface (Tuijthof et al., 2011), could be easily analyzed, as such detailing is essential to ascertain the tissue stiffness. This procedure helps the orthopedic surgeons make decisions regarding whether the meniscus should be repaired or resected (Anetzberger et al., 2014).

In our research, we utilized the menisci with no apparent signs of degeneration or injury. As patient-specificity is one of the notions of our envisioned digital twin for knee arthroscopic surgery (Bjelland et al., 2022), including patient demographics is a future consideration. Collagen fibers in degenerative tissues differ in composition and structure from those in healthy tissues (Hui Mingalone et al., 2018). Age also affects the collagen fiber’s extensibility (Nesbitt et al., 2021). This necessitates the tuning of parameters in the proposed model in accordance with the age and health condition of the patient. Moreover, the constitutive model should be modified to accommodate gender details, as all of these are crucial in developing a digital twin for a specific patient. However, the presented model would be adequate for studying generic meniscal tear mechanisms, designing meniscal repair procedures, simulating existing or innovative arthroscopic meniscal repair techniques, and designing implants.

## Data Availability

The raw data supporting the conclusion of this article will be made available by the authors, without undue reservation.
